# How to Improve Performance in Bayesian Inference Tasks: A Comparison of Five Visualizations

**DOI:** 10.3389/fpsyg.2019.00267

**Published:** 2019-02-20

**Authors:** Katharina Böcherer-Linder, Andreas Eichler

**Affiliations:** ^1^ Institute of Mathematics, University of Freiburg, Freiburg, Germany; ^2^ Institute of Mathematics, University of Kassel, Kassel, Germany

**Keywords:** epistemic uncertainty, Bayesian situations, judgment and decision making, visualization of statistical information, nested-sets structure

## Abstract

Bayes’ formula is a fundamental statistical method for inference judgments in uncertain situations used by both laymen and professionals. However, since people often fail in situations where Bayes’ formula can be applied, how to improve their performance in Bayesian situations is a crucial question. We based our research on a widely accepted beneficial strategy in Bayesian situations, representing the statistical information in the form of natural frequencies. In addition to this numerical format, we used five visualizations: a 2 × 2-table, a unit square, an icon array, a tree diagram, and a double-tree diagram. In an experiment with 688 undergraduate students, we empirically investigated the effectiveness of three graphical properties of visualizations: area-proportionality, use of discrete and countable statistical entities, and graphical transparency of the nested-sets structure. We found no additional beneficial effect of area proportionality. In contrast, the representation of discrete objects seems to be beneficial. Furthermore, our results show a strong facilitating effect of making the nested-sets structure of a Bayesian situation graphically transparent. Our results contribute to answering the questions of how and why a visualization could facilitate judgment and decision making in situations of uncertainty.

## Introduction

**Figure 1:**
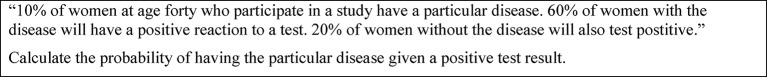
A medical context of judgment and decision making under uncertainty.

A typical case of judgment and decision making in a situation of epistemic uncertainty emerges when a medical diagnosis test yields a positive result. In this situation, the physician has to make a judgment and a decision about the health status of his or her patient and possibly about further treatment. An often cited example is shown in Figure 1 (cf. Johnson and Tubau, 2015, p. 3).

The uncertainty of the given situation is twofold. On one hand, medical diagnosis tests comprise an aleatory uncertainty, that is, an uncertainty that could not be changed in a given situation, similar to the probability distribution of dice. On the other hand, the uncertainty that a physician has concerning the health status of a patient represents epistemic uncertainty that is based on lack of knowledge (for both types of uncertainty, cf. Baraldi et al., 2014). Epistemic uncertainties can be changed by further information, such as a positive result on a diagnosis test, since the test result changes the physician’s knowledge status. For this reason, appropriately processing important information in a situation, such as a medical diagnosis test, is a crucial competence of professionals as well as laymen when confronted with epistemic uncertainty (e.g., Koller and Hoffrage, 2015). Similar judgments and decisions are also essential for lawyers, if evidence is given concerning a person being guilty or innocent (e.g., Satake and Murray, 2014), as well as in other professions (Hoffrage et al., 2015; Mellers et al., 2017). By contrast, a failure of processing information in a situation of epistemic uncertainty can lead to misjudgments and severe consequences (e.g., Stine, 1998; Schneps and Colmez, 2013). For this reason, the main aim of this paper is to contribute to answering the question of how to facilitate judgment and decision making in situations of epistemic uncertainty.

**Figure 2:**
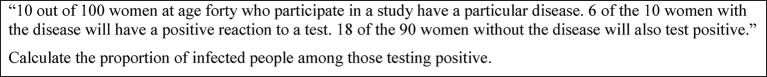
The medical context of Figure 1 with statistical information represented by natural frequencies.

A main model to process information in a situation of epistemic uncertainty is Bayes’ formula, as shown in Figure 1. This formula allows a quantitative judgment for one of the several possible hypotheses. Therefore, we call a situation as shown in Figure 1 a “Bayesian situation”. In our example, there are two possible hypotheses: having the disease or not. If H is the hypothesis and D the information, the epistemic uncertainty P(H) could be replaced by $P\left(H|D\right)=\frac{P\left(D|H\right)\cdot P\left(H\right)}{P\left(D|H\right)\cdot P\left(H\right)+P\left(D|\bar{H}\right)\cdot P\left(\bar{H}\right)}$. Unfortunately, both professionals and laymen often fail to process information in a Bayesian situation (Eddy, 1982; Hoffrage et al., 2000; Ellis et al., 2014). Based on the importance of appropriately dealing with epistemic uncertainties in different professions (see above), it has been reported that different strategies, such as using natural frequencies and using visualization, can greatly enhance performance in these situations (Gigerenzer and Hoffrage, 1995; Brase, 2009). A meta-analysis by McDowell and Jacobs (2017) found that the natural frequencies strategy increases participants’ performance from about 4% to about 24%. The representation of the situation in Figure 1 with natural frequencies is shown in Figure 2.

By contrast, discussion about a facilitating effect of visualizing statistical information in Bayesian situations is more ambiguous. Actually, it is an ongoing question which kind of visualization effectively increases people’s performance in Bayesian situations (e.g., Binder et al., 2015). Furthermore, it is an open question why visualizations are essential for improvement, or rather, which properties of the visualizations are essential for improvement (e.g., Brase, 2009, 2014; Sirota et al., 2014b). For this reason, the main aim of our study is to investigate properties of visualizations that could potentially increase people’s performance in Bayesian situations beyond the effect of natural frequencies; thus, the study could contribute to a prescriptive theory of improving statistically driven judgment and decision making in situations of epistemic uncertainty.

In this paper, we first discuss in detail visualizations of Bayesian situations and their possible facilitating effect, based on which we propose three hypotheses. These three hypotheses were investigated in an experiment that we conducted in a sample of 688 undergraduate students.

## Research Questions and Hypotheses Concerning Visualizing Statistical Information

In former research, three graphical properties of visualizations were considered to be beneficial in the context of boosting performance in Bayesian situations. The three main ideas are representing the statistical information area proportionally, using discrete objects, and making the nested-sets structure of a Bayesian situation transparent. In the following, we discuss these three approaches of boosting performance in Bayesian reasoning tasks. Performance was measured by the ability to solve Bayesian reasoning tasks in the frequency format (Figure 2). Based on our former research results (Böcherer-Linder and Eichler, 2017), this discussion leads to a research question and a related hypothesis for each of the three approaches.

**Figure 3:**
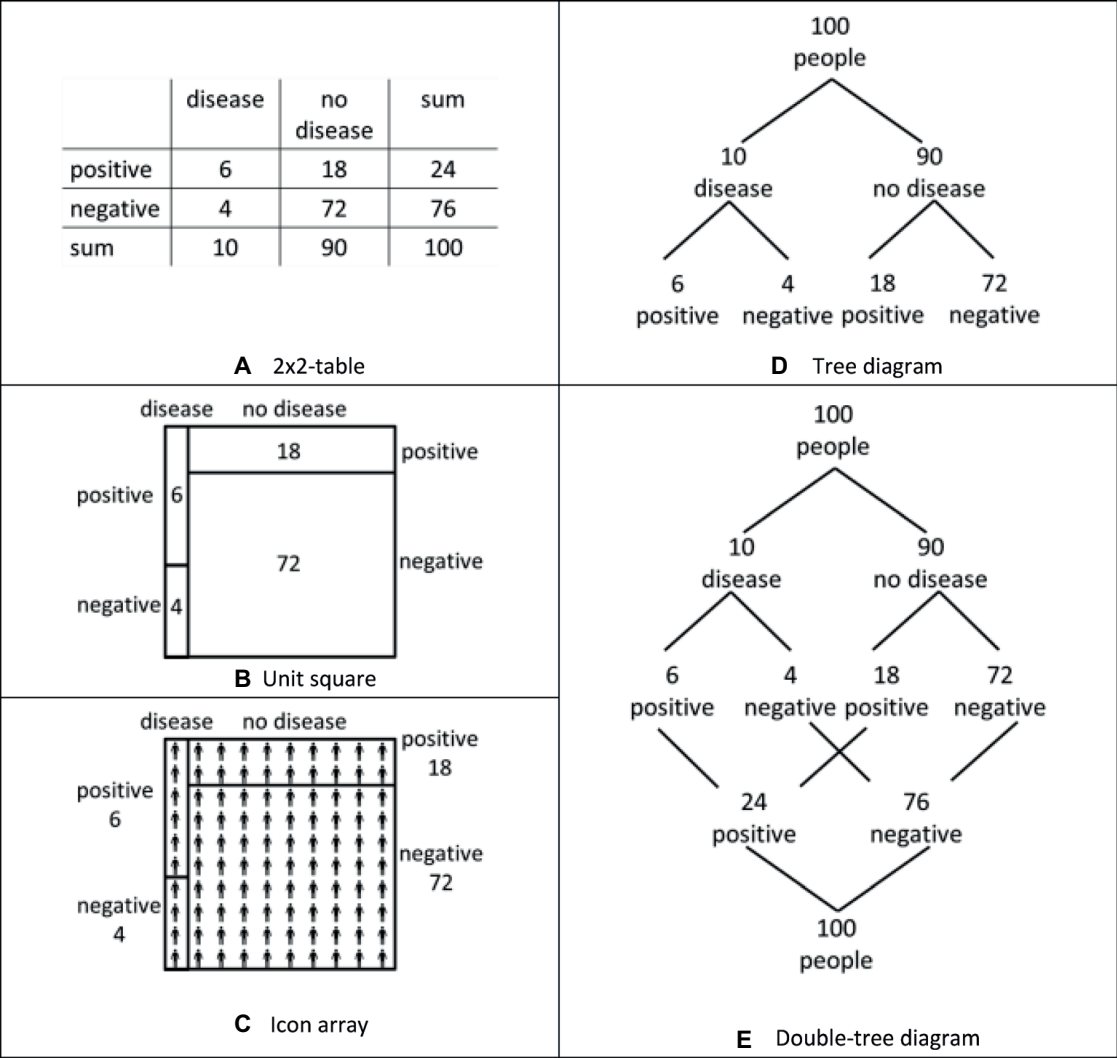
Five visualizations of the medical diagnosis situation, that is, a 2×2-table **(A)**, a unit square **(B)**, an icon array **(C)**, a tree diagram **(D)**, and a double-tree diagram **(E)**.

Starting with the 2 × 2-table that contains the information in terms stated as simply as possible, a unit square combines the properties of the 2 × 2-table with an area-proportionality (see Figures 3A,B). Second, the icon array combines the properties of the unit square showing discrete objects (Figure 3C). Finally, the double-tree diagram combines the properties of the tree diagram and the property of a graphical transparency of nested sets (Figures 3D,E).

### Area-Proportionality (Comparison of the 2 × 2-Table and the Unit Square)

One idea about beneficial graphical properties of visualization refers to representing the statistical information area proportionally (e.g., Tsai et al., 2011; Micallef et al., 2012): “this accurate, proportional representation is considered a key feature of what makes a good visual aid” (Talboy and Schneider, 2017, p. 375). Theoretical arguments for area-proportional visualizations, such as the unit square (Figure 3B), are often formulated based on mathematical considerations: “Rectangular areas correspond to probabilities and can be used to calculate their numerical value and to determine the Bayes relation” (Oldford, 2003, p. 1). Area-proportional visualizations increased performance in Bayesian reasoning tasks in Tsai et al. (2011), whereas area-proportionality did not prove to be a facilitating factor in Micallef et al. (2012) or Talboy and Schneider (2017).

We refer to the unit square (see Figure 3B), which was an effective visualization in our former research (Böcherer-Linder and Eichler, 2017) and which is an area-proportional visualization. In contrast to the unit square, the 2 × 2-table (see Figure 3A) is a visualization of the same graphical style (cf. Khan et al., 2015) without the property of area-proportionality. Therefore, by comparing the unit square with the 2 × 2-table, the first research question is whether the area-proportionality of the unit-square has an effect on performance in Bayesian reasoning tasks. Following the view of Talboy and Schneider (2017) and Oldford (2003), we thus hypothesize the following:

*Hypothesis 1:* A unit square will be more effective than a 2 × 2-table with respect to performance in Bayesian reasoning tasks.

### Discrete Objects (Comparison of the Unit Square and the Icon Array)

A second idea about beneficial graphical properties of visualizations refers to using representations of “real, discrete and countable” objects (Cosmides and Tooby, 1996, p. 33). This graphical property has been claimed to be helpful because it imitates the natural sampling situation and “help tap into the frequency coding mechanisms of the mind” (Brase, 2009, p. 369). The theoretical background of this approach is the ecological rationality account assuming that people perform better if the problem presentation resembles a real environmental situation (Gigerenzer, 2017). Realizations of visualizations with real, discrete, and countable objects include icon arrays (see Figure 3C for an example) and frequency grids. Beneficial effects have been observed for these kinds of visualizations, for example, in Sedlmeier and Gigerenzer (2001), Brase (2009, 2014), and Garcia-Retamero and Hoffrage (2013), but not in Sirota et al. (2014b, Experiment 1). Concerning iconicity, that is, the extent to which the icons resemble the represented objects, Sirota et al. (2014b) and Brase (2014) did not find any positive effect.

We refer again to the unit square that proved to be an effective visualization (Böcherer-Linder and Eichler, 2017). Following the idea of representing discrete objects to boost performance, we pose the research question of whether the beneficial effect of the unit square can further be enhanced by adding discrete objects into the fields, which led us to the design of the icon array as shown in Figure 3C. According to the theoretical considerations above, we expect a beneficial effect because of the additional discrete objects in the visualization:

*Hypothesis 2:* An icon array will be more effective than a unit square with respect to performance in Bayesian reasoning tasks.

### Graphical Transparency of Nested Sets (Comparison of the Tree Diagram and Double Tree Diagram)

A third idea about beneficial graphical properties of visualization refers to the transparent representation of the nested-sets structure of Bayesian situations. The theoretical background for this approach is the nested-sets account claiming that “any manipulation that increases the transparency of the nested-sets relation should increase correct responding” (Sloman et al., 2003, p. 302). Examples of graphical representations of a nested-sets structure or rather nested-sets relation include Euler diagrams (e.g., Sloman et al., 2003, p. 298), roulette-wheel diagrams (e.g., Yamagishi, 2003, p. 98), and unit squares (Figure 3B), which are close to visualizations called treemaps (Shneiderman, 1992) or identical to visualizations called mosaic displays (Friendly, 2002) or eikosograms (Oldford, 2003). Beneficial effects have been observed for these kinds of visualizations, for example, in Sloman et al. (2003) and Yamagishi (2003) but not in Brase (2009, 2014).

In our own research (Böcherer-Linder and Eichler, 2017), we argued that the tree diagram shows a weak graphical transparency of nested sets. The main argument for this assertion was that in the tree diagram, subset relations are generally visualized by connecting branches but that no branch exists connecting the subset and the set that are necessary to apply Bayes’ formula, for example, the subset “infected and tested positive” and the set “all tested positive” (see Figure 3D). As a consequence, performance was not as high as for a diagram with transparent nested sets (Böcherer-Linder and Eichler, 2017).

Following Khan et al. (2015), the tree diagram and the unit square that we compared in Böcherer-Linder and Eichler (2017) represent different styles of visualizations, that is, a *Branch style* and a *Nested style*. However, a tree diagram can also simply be transformed into a visualization with high transparency of nested-sets structure by adding the missing branches: in a double-tree diagram (Figure 3E), the set or node “infected” (24) is indeed connected with the subset or node “infected among those testing positive” (6). For this reason, it is possible to compare two visualizations of the same style that differs mostly concerning the transparency of the nested sets. Thus, the question arises whether a double-tree diagram is indeed more effective because of its graphical transparency of nested sets. Following the prediction of the nested-sets account (see above), we formulate our third hypothesis:

*Hypothesis 3:* A double-tree diagram will be more effective than a tree diagram with respect to performance in Bayesian reasoning tasks.

For our design, the following two comments are noteworthy: First, each of the five visualizations used in this study (Figure 3) showed beneficial effects in former research compared to no visualization at all (e.g., 2 × 2-table: Binder et al., 2015; Talboy and Schneider, 2016; unit square: Tsai et al., 2011; Talboy and Schneider, 2016; icon array: Brase, 2009, 2014; tree diagram: Sedlmeier and Gigerenzer, 2001; Binder et al., 2015; double-tree diagram: Wassner, 2004). Thus, each visualization we used is an effective visualization when used in that context. Second, the five visualizations show a missing “numerical equivalence” among the visualizations that could potentially impact the results. We discuss this aspect in the method section.

By testing these three hypotheses, we want to push forward the question of whether adding or reducing one of the three properties—“area-proportionality,” “discrete objects,” and “transparent nested sets”—to a specific visualization enhances or impedes performance. The selection of two visualizations for each of the three comparisons was based on the consideration of comparing visualizations that are graphically as similar as possible but differ in the property under consideration. The results of the study may shed light on the question of whether the design of a specific visualization could be further enhanced by referring to the graphical properties of “area-proportionality,” “discrete objects,” and “transparency of nested sets.” By this, we seek to contribute to the question of how judgment and decision-making processes might be improved in situations of epistemic uncertainty.

## Experiment

### Method

*Participants:* The participants were 688 undergraduates at the University of Kassel (Germany) and were enrolled in a course of mathematics education for primary schools. This course does not include the five visualizations (Figure 3) and the Bayes’ rule in the curriculum. We determined the approximate number of participants by *a priori* power analysis (G*Power) referring to a t-test (one-tailed) for our three directional hypotheses (*α* < 0.05, *β* > 0.8, and Cohen’s *d* < 0.3). The participants were randomly assigned to a 2 × 2-table (*N* = 147), a unit square (*N* = 150), an icon array (*N* = 146), a tree diagram (*N* = 125), and a double-tree diagram (*N* = 120). We had no control group, since former research showed the effectiveness of each of the five visualizations compared to no visualization (see “Experiment”). We collected the participants’ data in two waves. We checked if there were differences between the two samples but did not find significant results.

**Figure 4:**
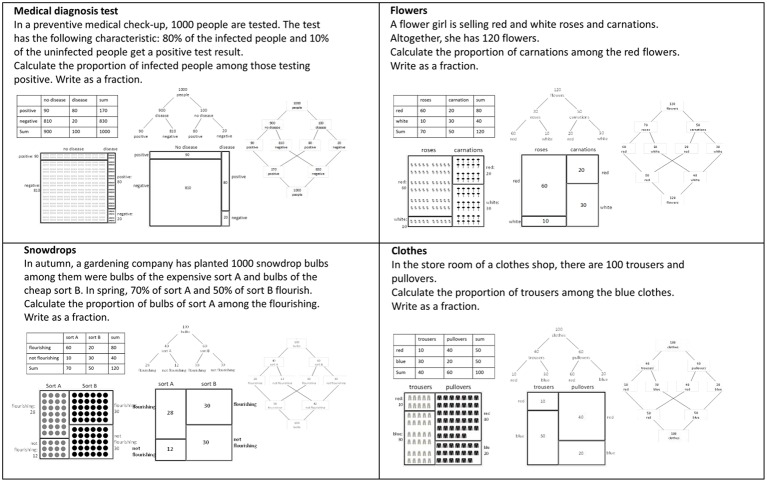
The test-items for investigating students’ performance when solving Bayesian reasoning tasks. The original test-items showed only one visualization.

*Materials and procedure:* To investigate the effectiveness of the five visualizations, we used five tests where the tasks, context stories, and the presented statistical data were the same, and only the visualizations differed. In Figure 4, we show the wording of the test items and the visualizations. Note that the original test items only showed one of the visualizations in each case. We chose a task format where we asked participants to calculate proportions and to write the solution as a fraction. Thus, we focused on applying Bayes’ formula and not on the interpretation of probability. Additionally, we decided not to give the natural frequencies in the text (except the total sample size) but only within the visualizations. Therefore, problems could only be solved by reading the information from the visualizations.

To introduce the visualizations, we gave a brief description of the visualizations on the front pages of the questionnaires. This description only explained how to read out simple information from the visualizations but not how to solve Bayesian reasoning problems. For the tree diagram and unit square, these descriptions were identical to Böcherer-Linder and Eichler (2017, p. 5) and were analogous for the double-tree diagram, 2 × 2-table, and icon array.

We designed these five visualizations (Figures 3, 4) with the idea that each of the visualizations has its own characteristics and we did not focus on numerical equivalence; for example, the 2 × 2-table represents the sums that are not shown in the unit square, and the double-tree diagram naturally bears more numerical information than the tree diagram. Since the participants had to read out the information from the visualizations, we additionally controlled whether each visualization was suitable for reading out simple information or sums over represented summands. Indeed, nearly every participant could answer questions such as, “How many people are not infected?” Additionally, the unit squares with column-sums (in Böcherer-Linder and Eichler, 2017) and without column-sums (in this research) showed similar effects. Therefore, we could exclude effects of the introductory description or of more or less numerical information within the visualizations.

In the icon arrays (Figure 4), we used icons with different degrees of iconicity (cf. Sirota et al., 2014b). The icon arrays of the items “Medical diagnosis test” and “Snowdrops” represent icons of low iconicity, and the icons of the items “Flowers” and “Clothes” represent icons of high iconicity. Since no effect of iconicity was observed in former research (Brase, 2014; Sirota et al., 2014b), we also did not expect any effect.

Since the participants were sitting close to each other when working on the tests, we arranged the items in different orders to avoid participants being influenced by each other. In our former research, where we used similar items (Böcherer-Linder and Eichler, 2017), we did not observe any effect from the order of the items. In this experiment, the seating arrangement of the participants and the order of the items was: unit square (flowers, diagnosis, clothes, snowdrops)–tree diagram (diagnosis, clothes, flowers, snowdrops)–icon array (flowers, diagnosis, clothes, snowdrops)–double tree (diagnosis, clothes, flowers, snowdrops)–2 × 2-table (snowdrops, flowers, diagnosis, clothes).

The experiment was carried out in accordance with the University Research Ethics Standards. Participation was voluntary without financial incentives, and anonymity was guaranteed. The data of our study are available at the Open Science Framework^1^.

### Results

**Figure 5:**
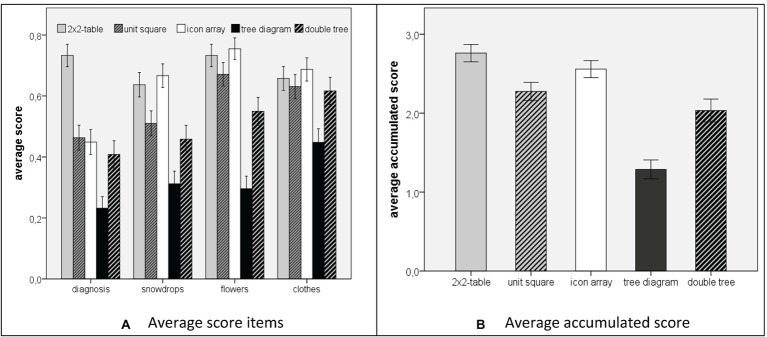
Participants performance when solving Bayesian reasoning tasks (**A**) score for single items; (**B**) accumulated score). The error bars indicate one standard error of the mean.

An answer was rated as correct when the proportion was equal to the exact value, that is, when the fraction had a correct numerator and denominator. Figure 5A illustrates the results for each of the four items and Figure 5B shows the accumulated score for the four items. Since the four items showed an acceptable reliability for each of the five visualizations (Cronbach’s Alpha: *α_2 × 2-table_* = 0.685; *α_unit square_* = 0.685; *α_icon array_* = 0.658; *α_tree_* = 0.695; *α_double tree_* = 0.810), we summarized over the four items in each case and investigated the hypotheses by comparing the accumulated scores (max = 4).

A Shapiro–Wilk test yielded a nonnormal distribution of the data. However, since the subgroups were large enough, we can assume robustness of the t-tests and of ANOVA with regard to nonnormality (Glass et al., 1972; Schmider et al., 2010). Therefore, we first conducted one-tailed t-tests to test the three hypotheses, which were directional and aimed at pairwise comparisons. Second, we applied an ANOVA for an additional exploratory analysis of our data.

Concerning *hypothesis 1*, we found a result contrary to the direction of the hypothesis. The students’ performance using the 2 × 2-table was 69.0% (*M* = 2.76, *SD* = 1.33), whereas their performance using the unit square was only 56.5% (*M* = 2.26, *SD* = 1.41). Therefore, the 2 × 2-table was significantly more effective than the unit square (*t*(293.619) = 3.142, two-tailed: *p* < 0.01, Cohen’s *d* = 0.37). Thus, *hypothesis 1* could not be confirmed. However, we found a significant result in the opposite direction.

Concerning *hypothesis 2*, the students’ performance was higher when the information was presented in the icon array (63.9% correct solutions, *M* = 2.56, *SD* = 1.31) compared to information presentation with a unit square (56.5%; *M* = 2.26, *SD* = 1.41). The difference was significant (*t*(294.238) = 1.882, *p* < 0.05) with a small effect (Cohen’s *d* = 0.22).

Concerning *hypothesis 3*, the percentage of correct solutions of students using the double tree (50.8%, *M* = 2.03, *SD* = 1.58) was significantly higher than the tree diagram (32.2%; *M* = 1.28, *SD* = 1.34, *t*(232.696) = 3.989, *p* < 0.001; Cohen’s *d* = 0.51). Thus, *hypothesis 3* was confirmed.

Since we administered the five visualizations in one sample of students, we further analyzed the relations between the performances in the five conditions in an exploratory way using an ANOVA that yielded a significant result, *F*(4) = 22.42, *p* < 0.001. Post-hoc *t*-tests (two-tailed) with a Bonferroni correction were significant concerning a comparison of the tree diagram with each of the other four diagrams, that is, with the double-tree diagram (*M* = 2.03; *SD* = 1.58, *t*(232.996) = 3.989; p* = 10*p* < 0.001 regarding the Bonferroni correction when testing 10 differences between two visualizations), with the 2 × 2-table (*M* = 2.76; *SD* = 1.33; *t*(261.881) = 9.980; p* < 0.001), the unit square (*M* = 2.26; *SD* = 1.41; *t*(268.553) = 5.824; p* < 0.001), and the icon array (*M* = 2.56; *SD* = 1.32; *t*(261.547) = 7.878; p* <0.001). The effect sizes (Cohen’s *d*) of the differences in participants’ performance to solve Bayesian reasoning tasks between the tree diagram and the other diagrams were mostly high (*d_tree/double-tree_* = 0.51; *d_tree/2 × 2-table_* = 1.25; *d_tree/unit-square_* = 0.82; *d_tree/icon-array_* = 1.08), which indicated that students’ performance was considerably lower when the information was presented in the tree diagram compared to each of the other four visualizations.

Post-hoc t-tests were significant concerning a comparison of the double tree diagram (*M* = 2.03; *SD* = 1.58) with the 2 × 2-table (*M* = 2.76; *SD* = 1.33; *t*(232.385) = 4.078; p* < 0.001) and the icon array (*M* = 2.56; *SD* = 1.32; *t*(231.089) = 2.959; p* < 0.05). These results indicated that students’ performance was lower when the information was presented in the double tree diagram compared to the 2 × 2-table and the icon array.

## Discussion

There is some research evidence that supports the claim that visualizations can have an additional beneficial effect on dealing with Bayesian situations beyond representing statistical information by natural frequencies (Garcia-Retamero and Hoffrage, 2013; McDowell and Jacobs, 2017). Since research results are ambiguous with regard to which specific properties of a visualization are beneficial, we investigated five visualizations that vary concerning their style (Khan et al., 2015), form, and, particularly, concerning three properties that were found to be potentially facilitating when dealing with Bayesian situations, that is, the area-proportional representation of the statistical information, the display of discrete and countable entities, and the graphical transparency of nested sets.

First, the comparison of the 2 × 2-table and the unit square yielded no additional beneficial effect of area proportionality. To the contrary, our results imply that the 2 × 2-table had a significant positive effect compared to the unit square with a small effect (Cohen’s *d* = 0.37). This result is interesting since area proportionality is the main graphical difference between the unit square and the 2 × 2-table, whereas both visualizations make the nested-sets structure transparent and show no countable objects. Our results are different from Micallef et al. (2012) who found no difference in performance between visualizations that were partly area-proportional and partly not. Additionally, in an intervention study, Talboy and Schneider (2017, p. 379), who compared the 2 × 2-table and a unit square in a training study, found that “those who were trained with graphs […] performed comparably overall with those who were trained with tables.” One explanation for the unexpected result in this study is the different degree of familiarity of the 2 × 2-table and the unit square. In German schools, the 2 × 2-table is a familiar visualization. Accordingly, 86% of the participants indicated knowing the 2 × 2-table. In contrast, only 34% of the participants indicated knowing a visualization like the unit square. An alternative explanation for the unexpected supremacy of the 2 × 2-table could be supposed concerning the context. Thus, the difference between the 2 × 2-table and the unit square seems to be influenced by the item “diagnosis” that had the most extreme distribution of the data in the 2 × 2-situation compared to the other three items. However, both assumptions need to be investigated in further research.

Second, the icon array outperformed the unit square. This result seems to be a consequence of visualizing countable and discrete entities (icons), since other potentially effective properties remained constant (area-proportionality and transparency of nested sets). This result is in accordance with Brase (2009) who found a positive effect when adding dots into Euler diagrams. However, taking into account the small effect (Cohen’s *d* = 0.22), we would not go as far as Brase (2014) in claiming that icon representations “are the most powerful pictorial technique currently known for facilitating correct Bayesian reasoning” (p. 93), since in our study, the effect of the graphical transparency of nested sets was higher than the effect of representing discrete objects.

Third, the double tree diagram outperformed the tree diagram. An explanation of this result is that the double tree diagram makes the nested-sets structure of a Bayesian situation graphically transparent in contrast to the tree diagram. The effect of making the nested-sets structure transparent was prominent in our results. Furthermore, the unit-square, the icon array, and the 2 × 2-table make the nested-sets structure graphically transparent by neighboring fields that have to be considered in a Bayesian situation (cf. Böcherer-Linder and Eichler, 2017). Accordingly, the ANOVA and post-hoc *t*-test showed two things. First, the difference between the performances in Bayesian reasoning was high with large effects, when a visualization made the nested-sets structure of a Bayesian situation transparent (except for the comparison of the tree diagram with the double tree diagram). Although the representation of discrete objects also yielded a positive effect, our results imply that the most powerful visualization of Bayesian situations is a visualization with natural frequencies that make the nested-sets structure of a Bayesian situation graphically and numerically transparent. The effect of making the nested-sets structure transparent was constant across visualizations representing different styles identified by Khan et al. (2015) and was further constant between two visualizations of the same style (tree diagram and double-tree diagram). For this reason, the beneficial effect of making the nested-sets structure in a Bayesian situation transparent seems to be very clear.

Properties of the sample and the tasks’ characteristics could have influenced our results. First, our sample consisted of university students. Thus, the results must be interpreted with this in mind, since intellectual ability seems to have an impact on performance in Bayesian situations (e.g., Johnson and Tubau, 2015), and particularly, spatial abilities might influence the effect of visualizations (Ottley et al., 2016). However, although the students were enrolled in a mathematics education course, these students’ affinity to mathematics was (on average) not high, since every primary teacher in Germany has to take courses in mathematics independent of ability or motivation to learn mathematics. Second, the context of the Bayesian situations and the wording of the tasks could have affected performance (e.g., Siegrist and Keller, 2011; Böcherer-Linder et al., 2018). Actually, the different contexts in the Bayesian situations that we used influenced performance. However, concerning the focus of this paper, that is, the beneficial effect of visualizations’ properties, in the Bayesian situations that we used, no interaction effect between visualization and context was found. Finally, the degree of familiarity seems to be a property of a given visualization that has to be taken into account. Further research could take differences in the mentioned properties of visualizations into account, which might also provide explanations for the significant differences between the double tree diagram and both the 2 × 2-table and the icon array.

The results that we present in this paper are based on the first beneficial strategy of representing statistical information as natural frequencies. In addition to the numerical transparency of nested sets, the second beneficial strategy refers to making the nested-sets structure of a Bayesian situation graphically transparent. The connection of both strategies resulted in a performance of about 60% in different Bayesian reasoning tasks. This is a considerable facilitating effect compared to the low performance of about 5% if the statistical information is represented by probabilities and without visual aids (cf. McDowell and Jacobs, 2017). Is this successful enough? Since dealing with Bayesian situations inappropriately, as a specific class of situations of epistemic uncertainty, could include severe consequences, for example, making inappropriate judgments and decisions in a medical diagnosis test or a jury verdict, a performance of about 60% should be increased further. For this reason, a further focus could be placed on investigating interventions that potentially further increase performance in Bayesian situations (Sirota et al., 2014a). For training studies, the question arises of which property of a visualization would yield short-run or even long-run success in dealing with Bayesian situations (cf. Sedlmeier and Gigerenzer, 2001).

Moreover, although our results imply that the icon array and the 2 × 2-table are more effective than the unit square and, particularly, more effective than the double tree diagram when performance in Bayesian situations is considered, further properties of the visualizations could be taken into account. For example, graphical differences might play a role when applying these visualizations in training. For example, if statistical information is given in text format and has to be visualized actively, the icon array, even with dots, is difficult to construct if a sample in a Bayesian situation is big and comprises, for example, 1,000 statistical entities. Furthermore, recent research has argued for a “distinction between Bayesian performance and Bayesian reasoning” (Vallée-Tourangeau et al., 2015, p. 3). In this sense, the ability to adequately judge the influence of a parameter change in a Bayesian situation (e.g., the base rate, sensitivity, and specificity in a medical diagnosis situation) could be understood as part of Bayesian reasoning (Böcherer-Linder et al., 2017). Therefore, it is an interesting question if a visualization’s property is beneficial beyond performance in Bayesian situations. We hypothesize that area-proportional visualizations could be more effective than visualizations without area-proportionality when people were asked to judge a parameter change in a Bayesian situation.

## Conclusion

For one graphical property of visualizations, area-proportionality, we could not observe any positive effect. However, additional icons yielded a positive, albeit smaller, effect. We finally showed that visualizations making the nested-sets structure of the Bayesian situation graphically transparent could improve performance in Bayesian reasoning tasks and, thus, the ability to deal with situations of epistemic uncertainty. Thus, based on our results, the most powerful property of a visualization of Bayesian situations was the graphical transparency of the nested sets structure in these situations. Our findings could inform the debate about beneficial graphical properties of visual representations of statistical information in Bayesian situations and could serve as an empirical foundation for designing interventions for improving judgment and decision making based on Bayesian reasoning for both professionals and laymen.

## Author Contributions

Both authors equally contributed to the research approach, to the realization of the research and the concept and writing of the paper.

### Conflict of Interest Statement

The authors declare that the research was conducted in the absence of any commercial or financial relationships that could be construed as a potential conflict of interest.

## Footnotes

^1^https://osf.io/g2wx7/
